# A case report of multi-compartmental lipoma of the hand

**DOI:** 10.1080/23320885.2018.1469988

**Published:** 2018-05-02

**Authors:** Maria A. Bocchiotti, Arianna B. Lovati, Loris Pegoli, Giorgio Pivato, Alessandro Pozzi

**Affiliations:** a Department of Reconstructive and Plastic Surgery, Città della Salute e della Scienza Hospital, University of Turin, Turin, Italy;; b Cell and Tissue Engineering Laboratory, IRCCS Galeazzi Orthopaedic Institute, Milano, Italy;; c Hand and Reconstructive Microsurgery Unit, Humanitas Pio X Clinic, Milano, Italy

**Keywords:** Lipoma, hand tumor, multi-compartmental mass

## Abstract

This report shows a multi-compartmental lipoma that occupied a vast area of the hand volar compartment, the distal aspect of the Parona’s space and infiltrated the dorsal compartment between the IV and V metacarpal bones. The mass was entirely removed through a dorsal approach, minimizing risks of neurovascular and tendinous lesions.

## Introduction

Lipoma is one of the most common soft tissue tumors of the body [1], but it is extremely rare in the upper extremity [2], in particular in the hand, where it represents the 8% of benign tumors [3]. In these cases, lipoma mainly develops in the thenar/hypothenar area rather than within the Parona’s space. Lipoma originates from mesenchymal preadipocyte cells and histologically is identical to normal fat tissue [2]. These lesions are characterized by a progressive enlargement and they can compromise the hand functionality. Giant lipoma sizing ≥50 mm should be removed due to the possibility of malignant transformation to sarcoma, despite the rare occurrence in the hand [4,5]. As reported by several reviews of the literature, lipomas infiltrating the deep layers of the hand are surgically approached and removed through the palmar aspect [6–11]. We present a case report of a giant asymptomatic lipoma of the deep palmar and dorsal space of the hand that was removed en bloc through a dorsal surgical approach.

## Case report

### Case presentation

The informed consent was obtained from the patient involved in this study according to the Institutional Review Board and all procedures were in accordance with the institutional and national ethical standards. The patient was a retired 70-year-old female with a past medical history of hypertension, hypercholesterolemia and cutaneous psoriasis that presented a prominent swelling in her left hand. The woman had a more than 10-year history of slow increasing painless mass involving both the dorsal and volar aspects of her left non-dominant hand. In 2006, she initially noticed the swelling in the central area of the palm, and then progressively enlarged in dimensions over a period of ten years. On physical examination, the mass occupied the deep palmar space between tendons and metacarpal bones and protruded into the IV inter-metacarpal space, the third finger web and the IV and V finger, respectively. The superficial palpation of the protruding lesion revealed an elastic mass while no deeper palpation was possible. The mass itself did not cause any functional impairment or neurovascular symptoms. The overlying skin was stretched and normal. The surgical indication was given on the basis of the recent growth of the mass and on the patient’s concerns upon the potential development of neurovascular symptoms or malignant transformation of the mass.

### Investigations

**Figure 1.**
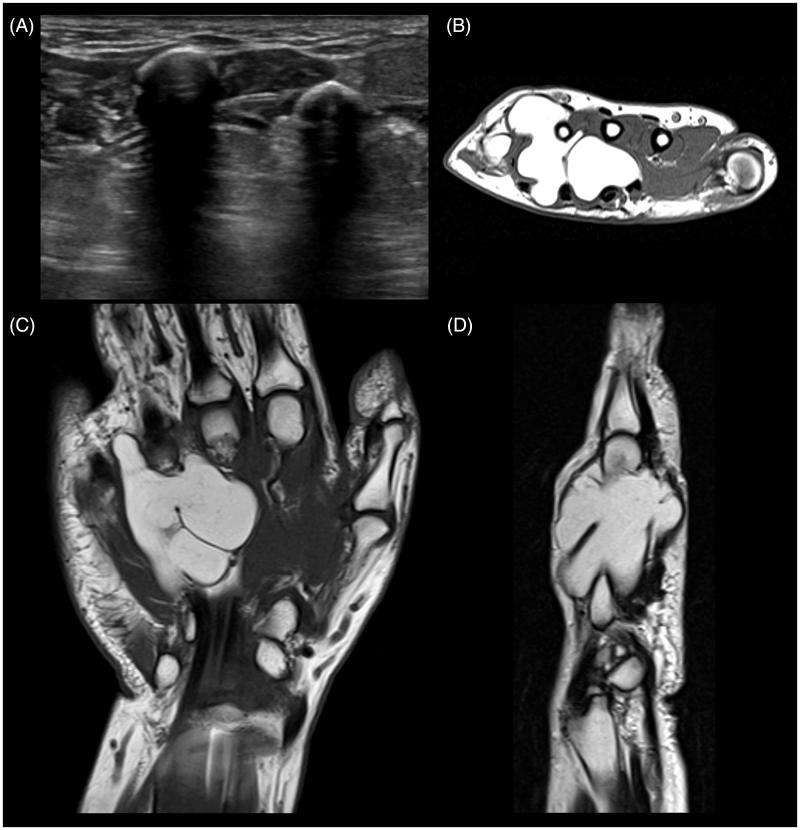
Diagnostic imaging of the tumor mass. (A) Ultrasound shows an encapsulated iso-hyperechoic avascular lesion extended dorsally from the II to the V metacarpal bone. (B) Axial, (C) coronal and (D) sagittal MRI images show a large hyperintense well-defined mass occupying the left hand recesses from the III to the IV.

On July 2010, ultrasounds showed a well-defined avascular hyperechoic mass of 19 × 10 × 8.5 mm within the superficial soft tissues of the dorsal left hand. The lesion lay superficially to the extensor tendon of the IV finger that presented a volar tenosynovitis sized 18 mm (data not shown). On February 2016, ultrasounds showed a well-defined iso-hyperechoic avascular lesion of 40 × 20 × 40 mm. It bridged the anterior and posterior compartments of the left hand (Figure 1(A)). In particular, the lesion extended both superficially from the II to the V metacarpal bone, involving interosseous muscles of the II, III and IV web spaces, and deeply to the flexor tendons from the II to the V space, involving the lumbrical muscles. The lesion reached the dorsal region of the hand within the IV web space with a size of 17 × 10 × 35 mm. The diagnosis was consistent with a giant lipoma. On February 2017, the MRI confirmed a multilobulated, encapsulated fatty mass with regular borders, thin vascular structures and some septal structures in the context. The hyper intense mass extended from the volar to the dorsal surface of the left hand involving the superficial cutaneous plans (Figures 1(B–D)). Particularly, such mass takes relationship of contiguity with the flexor tendons of the fingers, from the II to the V, with digitations penetrating the space between the tendons of the III and IV finger and between the tendons of the IV and V finger, in the absence of signs of invasiveness. On the dorsal space, it was observed a small digitation protruding into the third metacarpal space, and a greater portion that protruded through the IV metacarpal space associated to the dislocation of the extensor tendons of the IV and V finger and the effusion of the tendon sheaths. Moreover, the mass determined the dislocation of the palmar interosseous muscles, the lumbrical muscles, and the dorsal interosseous muscle of the V metacarpal space. The diagnosis was consistent with an infiltrating giant lipoma.

### Surgical procedure

**Figure 2.**
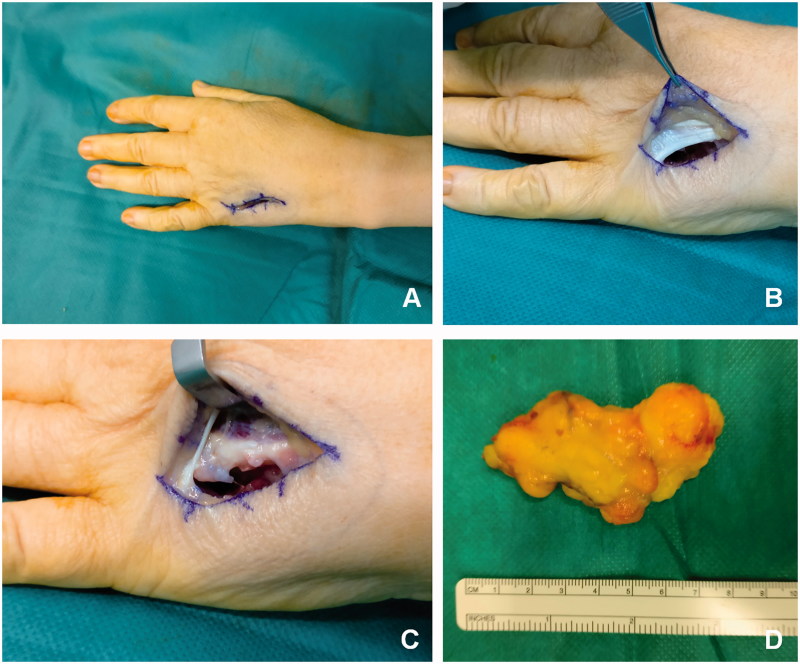
Surgical procedure and aspect of the **tumor** mass. (A) Curved skin incision over the IV inter-metacarpal space. (B) Inter-metacarpal ligament between the fourth and the fifth metacarpal bones. (C) The mass was pulled dorsally and dissected. (D) Lobular fatty mass after removal.

The surgery was carried out under axillary block and by tourniquet hemostasis. The mass was dorsally approached through a curved skin incision over the IV inter-metacarpal space (Figure 2(A)). The mass laid in the subfascial space, and after careful dissection from the dorsal interosseous muscles that were displaced by the tumor had been dissected from the volar compartment. The inter-metacarpal ligament between the fourth and the fifth metacarpal bones was left intact as there was enough space for the mass to be pulled dorsally and dissected (Figures 2(B,C)). No intramuscular infiltration was evident. Marginal excision of the lesion was performed and the encapsulated elliptical mass (70 mm length, 35 mm width) was removed en bloc from both the volar and dorsal compartments. It appeared as a lobular fatty mass (Figure 2(D)). Immediately after surgery, the hand was protected with a volar splint that left the metacarpal–phalangeal joint free to move. Ice was applied over the wound to prevent swelling and edema.

### Histopathology and post-operative course

Histopathology reported a well-encapsulated tumor composed of mature adipose cells consistent with a benign lipoma. No post-operative complications were experienced by the patients, only a mild swelling and hematoma occurred both in the palmar region and in the III, IV and V fingers. About 15 days after surgery, the stitches were removed and the patient presented only a mild functional impairment of the movements of the V finger due to the muscular atrophy induced by the lesion compression. After 10 months of follow-up the lesion did not present any sign of recurrence and any functional impairment has been shown by the patient.

## Discussion

This kind of lesions is commonly asymptomatic after the first diagnosis, however, it has been recommended to remove these masses despite asymptomatic in order to prevent complications, such as loss of strength or sensory damage [10]. The interesting aspect of this case relies on the choice of a solely dorsal approach to remove a multi-compartmental lesion of the hand. The dorsal approach represents a valid approach as it dramatically reduces the risk of neurovascular lesions of the ulnar bundle. The very nature of the mass that was still capsulated after 10 years from the first diagnosis allowing dissecting the mass from the dorsal approach without need of a second volar approach. A careful dissection is mandatory in these cases as if the mass is damaged or broken into several fragments a complete removal can be extremely hard from this approach. The view of volar compartment from the surgical field is not complete, and in the case of multiple fragments can be harder for the surgeon to identify and therefore remove every single piece of the mass. Thus, a combined dorsal and palmar approach should be intraoperatively considered in the presence of a fragmented mass or strict adhesion to the volar aspect of the hand with potential risks of neurovascular lesions. However, the dorsal approach reduced the post-operative pain compared to a double approach procedure, thus granting a faster recovery in the post-operative period due to lower swelling and scarring, and preventing the direct exposition and dissection of the ulnar neurovascular bundles and of the flexor tendons. This could explain why, in the present case, there was no reduction of both the grip and pinch measurement, while a strength reduction using a volar approach has been previously reported [4]. Finally, the natural history of the present case that remained asymptomatic and slowly growing for a decade represents a diversion from the majority of the hand-located lipomas, that naturally tend to have a faster grow-rate and can be rarely asymptomatic at the stage of the one discussed in the present report.
